# Trajectories of functioning in a population-based sample of veterans: contributions of moral injury, PTSD, and depression

**DOI:** 10.1017/S0033291720004249

**Published:** 2022-09

**Authors:** Shira Maguen, Brandon J. Griffin, Laurel A. Copeland, Daniel F. Perkins, Cameron B. Richardson, Erin P. Finley, Dawne Vogt

**Affiliations:** 1 San Francisco VA Health Care System, San Francisco, CA, USA; 2 University of California – San Francisco, San Francisco, CA, USA; 3 Central Arkansas VA Healthcare System, Little Rock, AR, USA; 4 University of Arkansas for Medical Sciences, Little Rock, AR, USA; 5 VA Central Western Massachusetts Healthcare System, Leeds, MA, USA; 6 University of Massachusetts Medical School, Worcester, MA, USA; 7 Pennsylvania State University, State College, PA, USA; 8 South Texas Veterans Health Care System, San Antonio, TX, USA; 9 University of Texas Health Science Center at San Antonio, San Antonio, TX, USA; 10 National Center for PTSD at VA Boston Health Care System, Boston, MA, USA; 11 Boston University School of Medicine, Boston, MA, USA

**Keywords:** Depression, functioning, moral injury, PTSD, veterans

## Abstract

**Background:**

Although research has shown that exposure to potentially traumatic and morally injurious events is associated with psychological symptoms among veterans, knowledge regarding functioning impacts remains limited.

**Methods:**

A population-based sample of post-9/11 veterans completed measures of intimate relationship, health, and work functioning at approximately 9, 15, 21, and 27 months after leaving service. Moral injury, posttraumatic stress, and depression were assessed at ~9 months post-separation. We used Latent Growth Mixture Models to identify discrete classes characterized by unique trajectories of change in functioning over time and to examine predictors of class membership.

**Results:**

Veterans were assigned to one of four functioning trajectories: *high and stable*, *high and decreasing*, *moderate and increasing*, and *moderate and stable*. Whereas posttraumatic stress, depression, and moral injury associated with perpetration and betrayal predicted worse outcomes at baseline across multiple functioning domains, moral injury associated with perpetration and depression most reliably predicted assignment to trajectories characterized by relatively poor or declining functioning.

**Conclusions:**

Moral injury contributes to functional problems beyond what is explained by posttraumatic stress and depression, and moral injury due to perpetration and depression most reliably predicted assignment to trajectories characterized by functional impairment over time.

## Introduction

In recent years, there has been a shift from focusing on psychological symptoms, disease, and disability to considering the full continua of physical, mental, and social well-being (Cooke, Melchert, & Connor, 2016). Although symptoms are important and can serve as indicators of well-being, functioning also contributes to well-being and is essential to assess in its own right, especially in the course of recovery from psychological trauma. To this end, studies that examine trajectories of posttraumatic stress (e.g. Galatzer-Levy, Huang,& Bonanno, 2018) or depression (e.g. Musliner, Munk-Olsen, Eaton, & Zandi, 2016) following exposure to stressful life events could be extended by studies that examine how functioning changes over time to improve understanding of recovery from psychological trauma.

Historically, scholars have linked potentially traumatic events (PTEs) to biological and behavioral fear responses endemic to posttraumatic stress disorder (PTSD). PTSD, especially when it is coupled with depressive symptoms, is associated with functional problems among veterans (Lippa et al., 2015). Yet, differences in psychological symptoms and functional problems may depend, at least in part, on the nature of the stressful event experienced by an individual. Moral injury is an important related construct that is potentially co-occurring but mechanistically distinct from PTSD and depression (Bryan, Bryan, Roberge, Leifker, & Rozek, 2018; Griffin et al., 2019). Events are considered morally injurious if they transgress deeply held moral beliefs and expectations, and the transgression shatters moral or ethical expectations that are derived from religious or spiritual beliefs, or cultural, organizational, and group-based rules (Litz et al., 2009). Exposure to potentially morally injurious events (PMIEs) may elicit lasting moral injury characterized by biological, psychological/behavioral, social, and religious/spiritual sequelae (Griffin et al., 2019).

Despite a growth of studies focusing on the psychological consequences of moral injury in the context of military service, few studies have explored associations between moral injury and functioning. One study that addressed this topic found that killing in war was associated with lower scores on a broad functioning composite that addressed functioning with respect to employment, education, physical health, and family problems with spouse or children (Maguen et al., 2009). Another empirical gap is the unknown contribution of moral injury to functional impairment, independent of mental health symptoms. For example, Maguen et al. (2012) found that even after adjusting for PTSD and depression, killing in war was uniquely associated with suicidal ideation, suggesting that moral injury might explain additional variance in negative outcomes, beyond its relationship with psychological problems.

Whereas these studies establish an association between moral injury and functional problems independent of other mental health symptoms, longitudinal investigations are needed to explore the contributions of moral injury, PTSD, and depression to changes in functioning over the course of recovery from psychological trauma. Better understanding these relationships can inform the development of novel treatments that focus on these shared difficulties and lead to more honed outreach efforts ensuring that individuals suffering with these issues get more targeted treatment.

The goal of this paper was thus to better understand the unique contributions of moral injury related to witnessing, perpetrating, and feeling betrayed, as well as posttraumatic stress and depression symptoms, to trajectories of intimate relationship, health, and occupational functioning among recently separated veterans. Based on prior research indicating that most veterans are resilient (e.g. Galatzer-Levy et al., 2018), we hypothesized that there would be a consistently high functioning trajectory which would comprise the majority of the sample, followed by a high and declining trajectory, a low and increasing trajectory, and a low trajectory that remained stable. Although there has been limited research on factors associated with how functioning changes across time, cross-sectional findings indicate that exposure to PMIEs is associated with functional deficits (e.g. Maguen et al., 2020), especially among those exposed to perpetration-based events who report guilt and shame, negative cognitions related to the self, and self-sabotaging behaviors, and those exposed to betrayal-based events that have difficulty trusting others in the future and feeling safety in relationships. For these reasons, after accounting for the contributions to PTSD and depression, we hypothesized that exposure to PMIEs by perpetration, betrayal, and to a lesser extent witnessing would be associated with a greater likelihood of functional problems, either initially or over time.

## Method

### Participants and procedure

Data were collected as part of a study of post-9/11 veterans' transitions from military to civilian life, The Veterans Metrics Initiative (TVMI) study (Vogt et al., 2018). This prospective cohort study drew from a roster of all separating US service members in fall 2016 identified from the VA/Department of Defense Identity Repository (VADIR). To be included in the sample, participants must have resided within the continental USA and separated from active-duty service or deactivated from the National Guard/Reserve following an activation of at least 180 days within 90 days prior to the first assessment occasion.

A modified Dillman outreach methodology was used, involving multiple contacts by mail and an opportunity to opt-out of additional contacts (Dillman, Smyth, & Christian, 2011). Potential participants were provided a link to a website where they could share their contact information and complete the survey. All potential participants received a pre-incentive of $5 cash, and those who completed surveys received an electronic gift valued at $20 at the first timepoint, with incentives increasing by $5 at each subsequent timepoint (Coughlin et al., 2011). Compared to the population of veterans who served in these most recent conflicts, with the exception that lower rank enlisted veterans were less represented, those who completed the initial survey were generally representative of the population in terms of demographic and military characteristics, with few meaningful differences in attrition across subgroups (Vogt et al., 2018). A consideration of how rates of probable PTSD and depression compared to other studies also suggests that the sample was generally representative of the larger population in terms of mental health. Specifically, average rates across the timepoints that were the focus of the current investigation (17.1% for PTSD; 17.8% for depression) were similar to the range of two other large-scale, representative studies that have examined veteran's mental health, which found rates of probable PTSD ranging from 13.8 to 23.2% and probable depression ranging from 13.7 to 16.4% (National Academies of Sciences, 2018; Schell & Marshall, 2008). However, probable PTSD was somewhat lower at the second wave, suggesting that wave 2 probable PTSD rates in this study may somewhat underestimate initial rates of probable PTSD.

Overall, 9566 participants responded at wave 1 (~3 months), 7200 at wave 2 (~9 months), 7201 at wave 3 (~15 months), 6480 at wave 4 (~21 months), and 5844 at wave 5 (~27 months). The current study utilized measures of moral injury, posttraumatic stress, and depression symptoms collected at wave 2. Psychosocial functioning was assessed at each assessment occasion; however, only data collected at waves 2, 3, 4, and 5 were analyzed for this study because changes were made to the functioning measures between waves 1 and 2. For these reasons, our analyses included only those who completed the wave 2 assessment occasion and any amount of the subsequent assessments. The number of participants with one (*n* = 364, 5.1%), two (*n* = 650, 9.0%), three (*n* = 1510, 21.0%) and four (*n* = 4676, 64.9%) valid responses indicated that the majority of participants completed at least three of four assessment occasions. The mean number of days between when participants completed wave 2 and wave 3 (*M* = 184.17 days, s.d. = 15.26 days), wave 4 (*M* = 359.29 days, s.d. = 15.03 days), and wave 5 (*M* = 543.88 days, s.d. = 13.88 days) showed that participants completed surveys without more than approximately 2 weeks deviation from the due date on average. Demographic and military-related characteristics are displayed in Table 1 (see also online Supplementary Table S1 for bivariate associations).

**Table 1.** Demographic and military-related characteristics (*N* = 7200)

Variable	Percentage
Gender	
Male	81.7%
Female	18.3%
Undisclosed/missing	0.0%
Age	
20–24.9	19.5%
25–29.9	22.4%
30–39.9	27.9%
40 +	28.3%
Undisclosed/missing	2.0%
Race/ethnicity	
Minority race/Eth.	33.9%
European American	66.1%
Undisclosed/missing	0.0%
Branch of service	
Army	37.8%
Navy	20.7%
Air Force	24.9%
Marines	16.6%
Undisclosed/missing	0.0%
Rank	
Officer/warrant	24.4%
Enlisted	75.6%
Undisclosed/missing	0.0%
Deployments	
0	29.2%
1	27.4%
2	16.5%
3 +	26.8%
Undisclosed/missing	0.1%
Positive PTSD screen	
Yes	7.5%
No	92.4%
Undisclosed/missing	0.1%
Positive depression screen	
Yes	17.5%
No	82.1%
Undisclosed/missing	0.1%

### Measures

The Well-being Inventory (WBI; Vogt et al., 2019) is designed to assess veterans' status, functioning, and satisfaction with regard to work, education, finances, health, and intimate relationships. The functioning scales for selected domains were used in this study: work (4 items; e.g. ‘You completed your work when expected’), health (12 items; e.g. ‘Completed recommended medical care’), and intimate relationships (6 items; e.g. ‘Provided your significant other with the emotional support they sought’). Participants rated each item using a response format from 1 = *never* to 5 = *most or all of the time*; items were averaged to create scores for each domain with higher scores indicating better functioning. Psychometric studies support the reliability and validity of the WBI among veterans (Vogt et al., 2019). Cronbach's *α* across timepoints for each domain was acceptable to strong: work (*α* = 0.82–0.85), health (*α* = 0.75–0.76), and intimate relationships (*α* = 0.88–0.89).

The Primary Care PTSD Screen (PC-PTSD-5; Prins et al., 2016) is a six-item self-report screening instrument on which participants utilized a dichotomous response format (yes/no) to indicate if they had been exposed to a traumatic event and, if so, whether they had experienced each of the Diagnostic and Statistical Manual of Mental Disorders, Fifth Edition (American Psychiatric Association, 2013) PTSD symptom domains within the past month (e.g. ‘Have you had nightmares about the event or thought about the event when you did not want to?’). Prins et al. suggest that three is the optimally sensitive cut-point, identifying 94.8% of participants who met criteria for PSTD using the MINI (Sheehan et al., 1998). We used the cut-point of three when reporting the percentage of the sample who screened positive for PTSD and a sum total score in regression analyses. Psychometric studies support the use of the PC-PTSD-5 among veterans (Prins et al., 2016). Cronbach's *α* in this sample was strong (*α* = 0.91).

The Patient Health Questionnaire-2 (PHQ-2; Kroenke, Spitzer, & Williams, 2003) is a two-item self-report screening instrument for depression occurring over a 2-week period (e.g. ‘Feeling down, depressed or hopeless’). Participants rated each item using a four-point response format (0 = *not at all* to 3 = *nearly every day*). Psychometric studies suggest that three is the optimally sensitive cut-point, identifying 87% of participants who met diagnostic criteria for depression using the Structured Clinical Interview for DSM-IV (Löwe, Kroenke, & Gräfe, 2005). We used the cut-point of three when reporting the percentage of the sample who screened positive for depression and a sum total score in regression analyses. Psychometric studies support the reliability and validity of the PHQ-2 among veterans receiving care in a Department of Veterans Affairs primary care setting (Corson, Gerrity, & Dobscha, 2004). Pearson's *r* in this sample indicated acceptable reliability (*r* = 0.80).

The Moral Injury Events Scale (MIES; Nash et al., 2013) was designed as a self-report assessment of exposure to and subjective distress stemming from potentially morally injurious military events. Nine items comprise the scale (e.g. ‘I am troubled by having acted in ways that violated my own morals or values’). Participants rated each item using a response format from 1 = *strongly disagree* to 6 = *strongly agree*. Although the proposed three-factor structure has had mixed support in research samples (Bryan et al., 2016; Richardson et al., 2020), conceptually the three-factor structure is most inclusive, consistent with our clinical experience, and provides a nuanced way to look at morally injurious events and their sequelae; the three factors assess moral injury stemming from witnessing, perpetrating or being betrayed. Estimates of internal consistency were acceptable for the two-item witnessing (*r* = 0.76), three-item betrayal (*α* = 0.82), and four-item perpetration (*α* = 0.93) subscales. We calculated a mean total score for each subscale.

### Data analysis

Preliminary analyses included missing data diagnostics as well as inspection of the variable distributions. First, we estimated a multiple regression with the total number of missing responses across all waves as the dependent variable [*F*_(7,7048)_ = 12.86, *p* < 0.001, *R*^2^ = 0.013], and gender, age, minority racial status, branch of service, and rank as independent variables. Being older (*β* = 0.10, *p* < 0.001) and a member of a racial minority group (*β* = 0.57, *p* < 0.001) was associated with more missing responses. Being a member of the Air Force in comparison to the Army was associated with fewer missing responses (*β* = −0.03, *p* = 0.045). For this reason, we concluded the data to be missing at random (Enders, 2010), and full information maximum likelihood was employed to maximize data available for analysis without biasing parameter estimates (Schafer & Graham, 2002). Next, inspection of the distribution of the functioning outcomes revealed some skewness (max = |1.67|) and kurtosis (max = |4.37|), which we accounted for by utilizing the Maximum Likelihood with Robust Standard Errors estimator (MLR). Analyses were performed using Mplus 8.2 (Muthén & Muthén, 1998–2017; syntax is available by request of the corresponding author).

For the main analyses, we employed Latent Growth Mixture Modeling (LGMM) to (1) identify distinct trajectories characterized by heterogeneous patterns of change over time in each functioning domain and (2) test whether class membership depended upon baseline ratings of posttraumatic stress, depression, and moral injury (van de Schoot, Sijbrandij, Winter, Depaoli, & Vermunt, 2017). First, by comparing a series of simple growth curve models using χ^2^ difference tests, we observed that a model with intercept (1,1,1,1), linear (0,1,2,3), and non-linear (0,1,4,9) growth parameters generally fit the data best. Residual variance in participants' observed functioning scores was constrained to be equal. Next, we regressed a latent categorical variable representing a specified number of latent trajectories (or classes) onto the growth parameters, obtaining particular intercept, slope, and quadratic parameters for each class. To determine the optimal number of classes, we compared a series of models with an iteratively greater number of classes. Model comparisons included the Lo-Mendell-Rubin Adjusted Likelihood Ratio Test (LRT) and Boot-Strapped Likelihood Ratio Test (BLRT). A significant *p* value on these indicates that a model with *k* classes fits the data better than a model with *k*–1 classes (Nylund, Asparouhov, & Muthén, 2007). Lower values for the Akaike Information Criterion (AIC), Bayesian Information Criteria (BIC), and sample size adjusted Bayesian Information Criteria (aBIC) were preferred (Jung & Wickrama, 2008). Entropy values closer to 1.00 indicated greater accuracy of classification (Lubke & Muthén, 2007). Finally, we inspected plots of the mean levels of functioning across time by class for each model, and we evaluated the percentage of the sample assigned to each class (Hipp & Bauer, 2006). In sum, optimal models were selected based on a preponderance of fit comparisons, theoretical coherence, and interpretability.

Finally, we added participants' ratings of posttraumatic stress, depressive symptoms, and moral injury stemming from witnessing, perpetrating, and being betrayed to the models as predictors of the latent variables. Consistent with Asparouhov and Muthén's (2013) recommendations, we estimated the growth parameters while including direct effects from the auxiliary variables. In order to facilitate model convergence, intercepts were freely estimated and residual variances for the linear and quadratic parameters were fixed (see also, deRoon-Cassini, Mancini, Rusch, & Bonanno, 2010). Next, measurement error for the latent categorical variable representing most likely class membership was determined. Finally, we estimated associations between the predictors and latent variable representing most likely class membership, with measurement error specified to the values computed in step two. Results predicting most likely class membership were obtained in the form of multinomial logistic regressions.

## Results

### Intimate relationship functioning

As is displayed in Table 2, review of the information criterion indices showed lower values with each additional class for models specifying one to six classes. However, the LRT indicated that the six-class solution fit no better than the five-class solution. The five-class solution was also not ideal because less than 1% of the sample was assigned to one of the classes. Inspection of the plotted estimated mean values of intimate relationship functioning across time revealed that the four-class solution contained trajectories that were different in form from trajectories revealed by models with fewer classes (online Supplementary Fig. S1), while the five-class solution split one of the primary trajectories observed in the four-class solution into two separate classes with minor differences. Entropy values indicated acceptable accuracy of class assignment for the four-class solution. We therefore selected the four-class solution as optimal.

**Table 2.** Fit indices for one to six class latent growth mixture models of functioning outcomes

Outcome	AIC	BIC	aBIC	LRT	BLRT	Entropy
Relationship functioning						
1-class	39 000.513	39 068.264	39 036.487			
2-class	38 657.702	38 752.553	38 708.064	*p* *<* 0.001	*p* *<* 0.001	0.96
3-class	38 362.635	38 484.586	38 427.387	*p* = 0.019	*p* *<* 0.001	0.92
4-class^a^	38 199.419	38 348.470	38 278.560	*p* *<* *0*.001	*p* *<* 0.001	0.72
5-class	38 055.979	38 232.132	38 149.510	*p* = 0.031	*p* *<* 0.001	0.74
6-class	37 929.576	38 132.829	38 037.496	*p* = 0.692	*p* *<* 0.001	0.75
Health functioning						
1-class	23 085.045	23 153.795	23 122.018			
2-class	22 677.169	22 773.419	22 728.930	*p* = 0.024	*p* *<* 0.001	0.91
3-class^a^	22 481.262	22 605.012	22 547.812	*p* = 0.021	*p* *<* 0.001	0.92
4-class	22 270.159	22 421.410	22 351.499	*p* = 0.007	*p* *<* 0.001	0.92
5-class	22 149.777	22 328.527	22 245.905	*p* = 0.114	*p* *<* 0.001	0.91
6-class	22 081.321	22 287.571	22 192.238	*p* = 0.160	*p* *<* 0.001	0.88
Work functioning						
1-class	32 221.208	32 289.288	32 257.511			
2-class	31 181.743	31 277.056	31 232.567	*p* *<* *0*.001	*p* *<* 0.001	0.90
3-class^a^	30 548.055	30 670.600	30 613.400	*p* *=* *0*.043	*p* *<* 0.001	0.89
4-class	29 990.807	30 140.585	30 070.674	*p* *=* *0*.407	*p* *<* 0.001	0.88
5-class	29 495.743	29 672.753	29 590.131	*p* = 0.002	*p* *<* 0.001	0.89
6-class	29 298.521	29 502.763	29 407.430	*p* = 0.444	*p* *<* 0.001	0.88

AIC, Akaike Information Criterion; BIC, Bayesian Information Criterion; aBIC, Sample Size Adjusted Bayesian Information Criterion; LRT, Lo-Mendel-Rubin Likelihood Ratio Test; BLRT, Bootstrapped Likelihood Ratio Test.

a Best-fitting model.

**Fig. 1.**
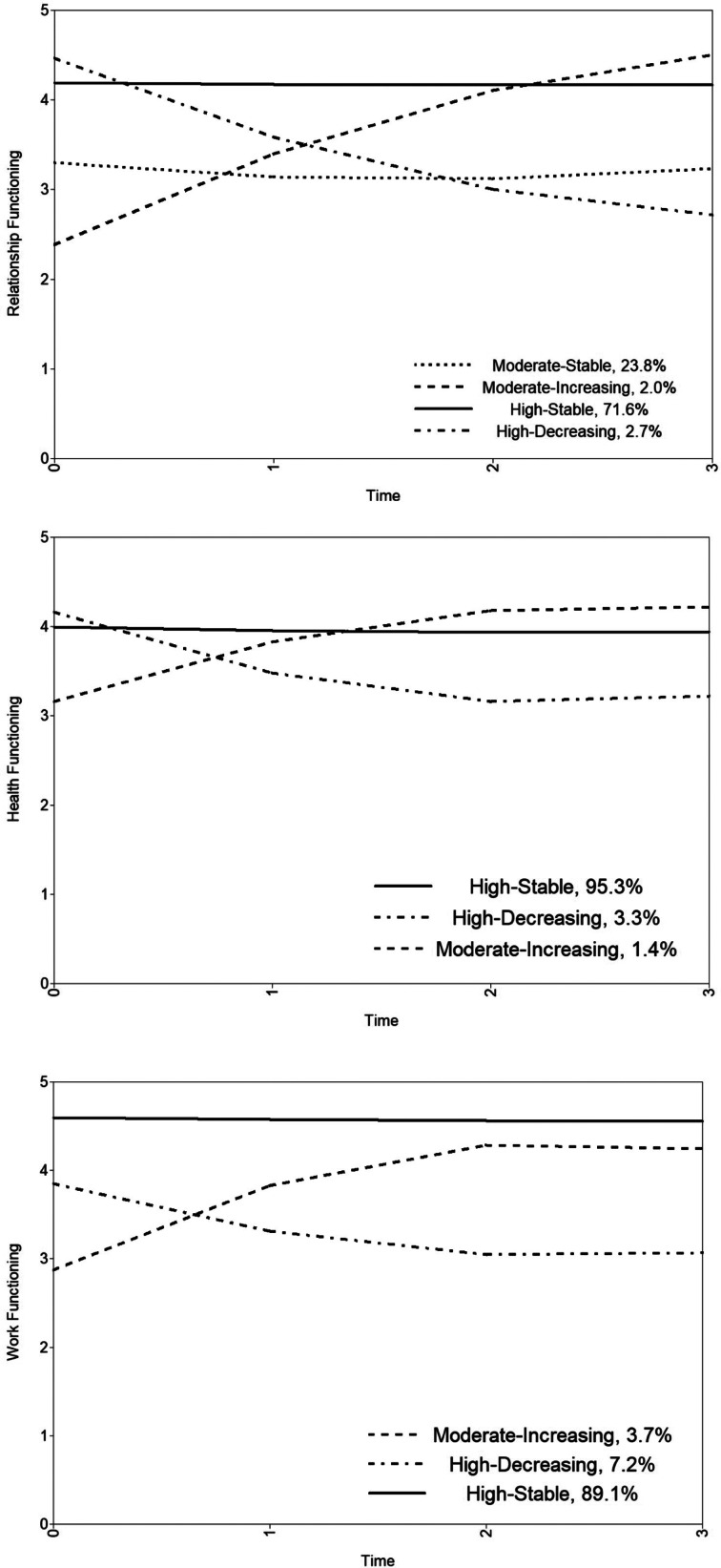
Estimated means by known class for relationship, health, and work functioning.

Class-specific intercept, slope, and quadratic growth parameters are reported in Table 3, and estimated means by known class are plotted in Fig. 1 (for observed individual values, see also online Supplementary Fig. S2). Veterans assigned to the first class (23.8%, *n* = 1538), which we labeled the *moderate-stable* class, exhibited a moderate level of intimate relationship functioning initially that was stable across time (Hedge's *g_rm_* = 0.04; Lakens, 2013). Next, a minority of the sample (2.0%, *n* = 129) was assigned to the *moderate-increasing* class, characterized by a moderate level of intimate relationship functioning initially that increased with time (Hedge's *g_rm_* = 3.02). The majority of the sample (71.6%, *n* = 4633) was assigned to the *high-stable* class and reported a high level of intimate relationship functioning initially that was maintained over time (Hedge's *g_rm_* = 0.02). Finally, veterans (2.7%, *n* = 172) whose intimate relationship functioning was initially high and declined with time were assigned to the *high-decreasing* class (Hedge's *g_rm_* = 2.40).

**Table 3.** Growth parameters by known class

	Relationship Functioning		Health Functioning		Work Functioning	
Classes	Est.	S.E.	Est.	S.E.	Est.	S.E.
Class 1						
Intercept	3.68***	0.048	4.29***	0.011	3.19***	0.147
Slope	−0.23***	0.041	−0.05***	0.006	1.20**	0.368
Quadratic	0.07***	0.013	0.01***	0.002	−0.25**	0.095
Class 2						
Intercept	2.91***	0.156	4.44***	0.050	4.16***	0.114
Slope	1.17***	0.279	−0.87***	0.216	−0.68***	0.156
Quadratic	−0.15	0.097	0.19*	0.076	0.14**	0.041
Class 3						
Intercept	4.52***	0.026	3.57***	0.114	4.79***	0.013
Slope	−0.03	0.014	0.83***	0.183	−0.02*	0.009
Quadratic	0.01	0.004	−0.16**	0.060	0.003	0.003
Class 4						
Intercept	4.77***	0.063	–	–	–	–
Slope	−1.03***	0.093	–	–	–	–
Quadratic	0.15	0.093	–	–	–	–

Est., estimate; S.E., standard error.

*p < 0.05, **p < 0.01, ***p < 0.001.

As shown in Table 4, direct effects of the auxiliary variables on the intercept parameter (class-invariant), revealed that higher levels of posttraumatic stress, depression, and moral injury stemming from perpetration and betrayal (but not witnessing) were associated with poorer intimate relationship functioning initially. We then explored associations between the auxiliary variables and class assignment (see Table 5). Higher depression ratings at baseline were associated with greater odds of membership in the *moderate-stable* and *moderate-increasing* classes relative to the *high-stable* class. Higher levels of moral injury stemming from perpetration were associated with greater odds of membership in the *high-decreasing* and *moderate-increasing* class relative to the *high-stable* class. Veterans who reported higher levels of moral injury stemming from betrayal had higher odds of being assigned to the *high-stable* class relative to the *high-decreasing* class (perhaps because veterans assigned to the *high-stable* class on average reported lower levels of intimate relationship functioning when compared to those assigned to the *high-decreasing* class at baseline but no other assessment occasion, see Fig. 1). Veterans reporting higher levels of moral injury stemming from betrayal also had greater odds of being assigned to the *moderate-stable* class than the *high-decreasing* class (OR 1.57, *p* = 0.005, 95% CI 1.22–2.03) and marginally greater odds of being assigned to the *moderate-stable* class than the *high-stable* class (OR 1.10, *p* = 0.059, 95% CI 1.00–1.20).

**Table 4.** Regression of intercept growth parameter on covariates

	Relationship Functioning		Health Functioning		Work Functioning	
Covariate	Est.	S.E.	Est.	S.E.	Est.	S.E.
Posttraumatic stress	−0.034***	0.009	−0.013**	0.005	−0.014*	0.007
Depression	−0.165***	0.007	−0.142***	0.004	−0.081***	0.006
Moral injury – Witness	0.005	0.006	0.009*	0.004	0.000	0.004
Moral injury – Perpetration	−0.046***	0.009	−0.038***	0.005	−0.038***	0.007
Moral injury – Betrayal	−0.030***	0.008	−0.036***	0.005	−0.016**	0.005

Est., estimate; S.E., standard error.

*p < 0.05, **p < 0.01, ***p < 0.001.

**Table 5.** Multinomial logistic regressions predicting class membership

	Posttraumatic stress		Depression		Moral injury – Witness		Moral injury – Perpetration		Moral injury – Betrayal	
	OR	95% CI	OR	95% CI	OR	95% CI	OR	95% CI	OR	95% CI
Relationship functioning										
High and decreasing	0.12	0.01–3.72	0.91	0.72–1.16	1.13	0.91–1.40	1.35*	1.05–1.73	0.70*	0.55–0.89
Moderate and increasing	0.82	0.64–1.06	1.26***	1.10–1.46	1.15	0.94–1.40	1.36**	1.12–1.66	0.92	0.73–1.15
Moderate and stable	1.02	0.93–1.11	1.20***	1.13–1.28	0.95	0.88–1.03	1.02	0.92–1.12	1.10	1.00–1.20
Health functioning										
High and decreasing	0.88	0.69–1.14	0.74*	0.56–0.97	1.06	0.87–1.28	1.29*	1.07–1.55	1.01	0.81–1.26
Moderate and increasing	0.94	0.72–1.22	1.04	0.86–1.26	0.97	0.76–1.22	1.66*	1.22–2.25	0.98	0.75–1.38
Work functioning										
High and decreasing	1.08	0.95–1.21	1.35***	1.22–1.48	1.06	0.92–1.22	1.35***	1.17–1.57	1.05	0.91–1.22
Moderate and increasing	1.05	0.96–1.14	1.35***	1.26–1.44	1.11*	1.02–1.22	1.16**	1.05–1.29	1.01	0.91–1.11

Odds Ratios (OR) with 95% Confidence Intervals (95% CI) represent the change in odds of assignment to each comparison class relative to the reference class (High and Stable Functioning) per unit increase in each predictor.

*p < 0.05, **p < 0.01, ***p < 0.001.

### Health functioning

Next, we examined veterans' health functioning scores. Review of the information criterion indices showed lower values for models containing an additional class for solutions specifying one to six classes. The LRT indicated that neither the six-class nor five-class solutions were better than simpler models containing one less class. Guidance from the entropy values indicated that the accuracy of class assignment decreased with the specification of additional classes beyond the three-class solution. Inspection of the plotted mean levels of health functioning across time revealed that the three-class solution contained distinct trajectories, while the four-class solution contained an additional class with a minor deviation from one of the three primary trajectories (online Supplementary Fig. S3). Thus, we selected the three-class solution as optimal.

Class-specific intercept, slope, and quadratic growth parameters are reported in Table 3, and estimated means by known class are displayed in Fig. 1 (for observed individual values, see also online Supplementary Fig. S4). The majority of the sample was assigned to the first class (95.3%, *n* = 6814) and reported high initial levels of health functioning that were stable over time (Hedge's *g_rm_* = 0.11), establishing the *high-stable* class. Next, a considerable minority (3.3%, *n* = 238) was assigned to the *high-decreasing* class, characterized by a high level of health functioning initially that declined over time (Hedge's *g_rm_* = 1.83). The third class (1.4%, *n* = 99), which we labeled as the *moderate-increasing* class, exhibited a moderate level of health functioning initially that improved with time (Hedge's *g_rm_* = 2.17).

Higher levels of posttraumatic stress, depression, and moral injury stemming from perpetration and betrayal were associated with poorer health functioning at baseline, and higher levels of moral injury from witnessing were associated with better health functioning at baseline (see Table 4). As is shown in Table 5, higher levels of moral injury from perpetration were associated with greater odds of membership in the *high-decreasing* class and *moderate-increasing* relative to the *high-stable* class. Higher depression scores at baseline were associated with lower odds of membership in the *high-decreasing* class relative to the *high-stable* class (again with the *high-stable* class on average reporting lower levels of health functioning when compared to those assigned to the *high-decreasing* class at baseline but no other assessment occasion, see Fig. 1).

### Work functioning

Finally, we examined veterans' work functioning. The information criterion indices indicated that fit improved with the specification of an additional class for models specifying one to six classes. Guidance from the LRT suggested that neither the six-class model nor the four-class model fits the data any better than models specifying one less class. Thus, either the three- or five-class solutions appeared best. The three- and five-class solutions demonstrated equivalent classification accuracy according to the entropy values. To adjudicate these results, we inspected plots of the mean values of work functioning across time (online Supplementary Fig. S1), and we examined the percentage of the sample assigned to each class. Whereas the three-class solution revealed trajectories with differing form and larger sample sizes by class, the five-class solution extracted additional classes that were only slightly different in form and contained few veterans (online Supplementary Fig. S5). We therefore selected the three-class solution as optimal.

Class-specific intercept, slope, and quadratic growth parameters are reported in Table 3, and estimated means by known class are displayed in Fig. 1 (for observed individual values, see also online Supplementary Fig. S6). The first class (3.7%, *n* = 249), which we labeled as the *moderate-increasing* class, exhibited moderate levels of work functioning initially that improved with time (Hedge's *g_rm_* = 2.10). Next, a considerable minority of the sample (7.2%, *n* = 478) was assigned to the *high-decreasing* class, reporting a high initial level of work functioning that decreased with time (Hedge's *g_rm_* = 0.98). Finally, the majority of the sample (89.1%, *n* = 5960) was assigned to the *high-stable* class, which was characterized by a high level of work functioning initially that was maintained over time (Hedge's *g_rm_* = 0.17).

Higher levels of posttraumatic stress, depression, and moral injury from perpetration and betrayal were associated with poorer initial levels work functioning (see Table 4). Nevertheless, only higher levels of depression and moral injury from perpetration were associated with greater odds of membership in the *high-decreasing* and *moderate-increasing* classes relative to the *high-stable* class (see Table 5). Higher levels of moral injury from witnessing were associated with greater odds of membership in the *moderate-increasing* class relative to the *high-stable* class.

## Discussion

Consistent with prior studies that have examined the trajectories of posttraumatic stress among adults exposed to PTEs (e.g. Donoho, Bonanno, Porter, Kearney, & Powell, 2017; Galatzer-Levy et al., 2018), we found distinct functioning trajectories among veterans following separation from military service. These trajectories include high initial levels of functioning that were maintained across time, moderate initial levels of functioning that were maintained across time, high initial levels of functioning that declined with time, and moderate initial levels of functioning that improved with time. In this population-based sample, most veterans maintained a relatively high level of functioning after separation from military service in intimate relationship, health and work functioning; however, notable proportions of the sample reported difficulties either initially or as time progressed. Functional problems during the first few years post-military depended in part on the types of psychological distress reported when separating from service. Depression in particular, more so than PTSD, was a strong and consistent predictor of initial deficits, with some reporting difficulties that persisted over time and others reporting recovery to initial levels of functioning. Interestingly, prior literature that has explored the impact of both PTSD and depression on functioning has found varying results, depending on the age group and type of functioning assessed. It is rare that PTSD and depression are examined in relationship to multiple functional outcomes, so this is a novel component of this study. More specifically, some studies have found that PTSD and depression equally impact functioning (e.g. disability in older veterans; Smith et al., 2020), PTSD impacts functioning more than depression (educational functioning in student veterans; Morissette et al., 2019), or depression impacts functioning more than PTSD (physical health functioning in service members; Asnaani et al., 2018). Future studies are needed to better understand which types of functioning are most impacted by depression.

Moral injury stemming from perpetration was consistently associated with worse functioning, more so than moral injuries from witnessing or betrayal, even after accounting for the impact of posttraumatic stress and depression. This is consistent with prior studies finding that killing in war, especially involving civilian women and children, is associated with poorer post-military outcomes (e.g. Maguen et al., 2009). Veterans with moral injury due to killing may self-sabotage their well-being due to beliefs that they do not deserve supportive relationships, healthy lifestyles, or desirable jobs (Litz et al., 2009; Purcell, Koenig, Bosch, & Maguen, 2016). We extend prior findings by using a longitudinal framework to demonstrate that those with moral injury stemming from perpetration may have had initial high levels of functioning that then declined over time in relationship, health, and work domains. There is evidence that the impact of moral injury may unfold over time and that coming to terms with integrating what happened in war with one's current identity can be a process (Purcell et al., 2016).

Although treatments are emerging that deal with the direct impact of killing in war (e.g. Burkman, Purcell, & Maguen, 2019; Maguen *et al*., 2017; Purcell, Burkman, Keyser, Fucella, & Maguen, 2018a, 2018b) and moral injury more generally (Gray et al., 2012; Harris et al., 2018; Norman, Wilkins, Myers, & Allard, 2014), assessment of exposure to PMIEs is still not part of routine clinical care (Maguen & Burkman, 2013). It is important that clinicians assess for PMIEs and moral injury in a thoughtful and sensitive manner and feel well-trained to do so. Given that some clinicians may feel that moral injury assessment and treatment go beyond their areas of expertise, it may be beneficial to allocate additional resources for training in moral injury and associated areas (e.g. spirituality). Overall, preventive efforts aimed at facilitating positive post-deployment functioning may therefore be improved by clinical assessment and treatment of moral injury. This is especially critical given the association between killing and suicide, even after accounting for a number of mental health outcomes (Maguen et al., 2012).

Veterans may also be distressed by exposure to transgressive acts for which others are responsible, either by witnessing or being betrayed. In the current study, betrayal-based moral injury was associated with worse relationship, health, and occupational functioning initially, although betrayal was not the strongest driver of functional problems over time when accounting for depression and PTSD. An area for continued examination is how moral injuries driven by others can impact interpersonal functioning and seeking care. For example, recent studies found a link between betrayal, and poorer health status (e.g. Klest, Tamaian, & Boughner, 2019), which appeared to depend in part on non-adherence to medical treatment and distrust toward providers.

Another important implication of this study relates to the conceptualization of moral injury following exposure to transgressive events for which others are responsible. Namely, a variety of factor structures have been proposed for the MIES (Bryan et al., 2016; Nash et al., 2013; Richardson et al., 2020), some that aggregate witnessing and being betrayed and others that separate the two. Findings from this study support disaggregating betrayal and witnessing as evidenced by divergent patterns of association between witnessing and betrayal with functional outcomes. For instance, betrayal was associated with poorer health functioning initially, and witnessing was associated with greater health functioning initially. Those who witnessed were also more likely to report work functioning that improved with time as opposed to stable and high work functioning. Additional psychometric work may be needed to better capture the aspects of moral injury by witnessing, especially among individuals, who despite providing excellent care, are unable to prevent others from suffering the inevitable injuries of war (e.g. combat medics, mortuary affairs, chaplains).

By better understanding how moral injury contributes to changes in psychosocial functioning during the process of post-deployment reintegration, we can hone assessment, and treatment to aid those at highest risk of adverse outcomes over time. More specifically, these trajectories could contribute to better tracking of functional outcomes over time as part of the treatment process, better measurement of unique post-deployment mental health issues (including PMIEs), and subsequently to more targeted treatment. For example, we know that those that have exposures to potentially morally injurious perpetration events have poorer functioning outcomes, yet these exposures are not systematically measured when a veteran engages in treatment, nor are functional outcomes. Veterans with these exposures can be referred to more targeted moral injury treatment that specifically targets functioning rather than referred to a general PTSD treatment that may not hone-in on the most problematic exposures, which can be missed within the PTSD framework.

Finally, prior research has found that morally injurious experiences are associated with profound guilt and shame (Griffin et al., 2019), which are often considered hallmark symptoms of moral injury and may act as mediators between exposures and functional impairment (Norman et al., 2018). It may also be that perpetration, in particular, compared to witnessing or betrayal, is associated with higher guilt and shame, which may explain some of our findings. While many veterans are exposed to PMIEs, not all go on to develop functional impairment, so it is important to better understand some of the mediators that may contribute to this impact. In addition to guilt and shame, other studies have found that factors such as self-forgiveness, meaning-making, and a sense of entrapment can all be associated with adverse outcomes (Currier, Holland, & Malott, 2015; Levi-Belz & Zerach, 2018; Purcell et al., 2018a, 2018b) and these mediators should continue to be explored in the context or moral injury.

### Limitations and future directions

Limitations of these findings are important to note. First, the percentages of the sample assigned to each class and the severity of reported distress may differ in other samples. In a large sample where most individuals function well, identifying chronically low functioning trajectories is difficult given the small number of individuals assigned to this group. Studies of treatment-seeking veterans may reveal more nuanced differences between trajectories (e.g. whether decline is early or delayed) and increase proportions of the sample assigned to classes characterized by impairment, thereby enhancing the utility of multinomial logistic regressions predicting class membership using additional variables (e.g. demographic and military-related characteristics). Second, results may differ in veterans of earlier eras. Specifically, health functioning in this sample was generally high, given that participants were primarily young and healthy veterans of more recent conflicts in Afghanistan and Iraq. Moreover, though the trajectories of relationship and work functioning identified using the full sample replicated in split half subsamples comprised of randomly selected cases, trajectories of health functioning replicated in one randomly selected subsample (i.e. high and stable, high and decreasing, moderate and increasing) but not another. In the other subsample, those assigned to the high and decreasing class were split according to whether impairment in health functioning was immediate or delayed. For this reason, replication studies that examine health functioning are especially important, including among veterans from combat eras earlier than the post-9/11 wars, because the downstream effects of stress-related issues may be delayed and perhaps more evident in older individuals. Third, the emerging literature on moral injury is still wrought with psychometric challenges, and further psychometric work is needed to develop a measure of morally injurious outcomes. Ideally, instruments measuring moral injury could clearly distinguish between exposures to PMIEs, including types, frequency and intensity of exposures, as well as emotional, cognitive, and behavioral manifestations of moral injury. Establishing a gold-standard clinical interview as well as self-report measure with specific cutoff scores to distinguish those with and without moral injury can assist with collecting more targeted and precise information about moral injury exposures and outcomes. Fourth, use of the PHQ-2 and other screening measures to capture mental health symptoms, rather than gold-standard clinical interviews or more comprehensive measures, could have introduced bias and future studies should employ the latter to add to the literature. Fifth, although the average rates of probable PTSD and depression fall within the range of other representative studies, rates of probable PTSD were somewhat lower in the second wave. While this could reflect a combination of both recovery over time as well as the fact that other studies were cross-sectional, it remains possible that wave 2 probable PTSD rates may somewhat underestimate initial rates of probable PTSD.

Given the dynamic process of both mental health symptoms as well as moral injury, further research is needed. Future studies should explore how moral injury unfolds over time, given that most studies examining moral injury have been cross-sectional. More specifically, it is important to better understand at what point after the PMIEs moral injury is most likely to emerge, whether there can be delayed moral injury, especially as veterans adjust to their post-deployment lives, and whether moral injury is a stable construct or tends to fluctuate over the life course (as well as any factors associated with these potential fluctuations).

## Conclusion

In this population-based, longitudinal sample of post-9/11 veterans, we found four classes of relationship, health, and work functioning, with the majority falling in the high and stable functioning class across each domain. Whereas posttraumatic stress, depression, and moral injury stemming from perpetration and betrayal predicted worse functioning outcomes at baseline, moral injury due to perpetration and depression most reliably predicted assignment to trajectories characterized by functional impairment over time. Moral injury related to witnessing and betrayal were less frequent predictors of poor functioning when accounting for mental health and other moral injuries. Additional research is needed to understand the relationship between moral injury and functioning, especially as it relates to suicide-related behavior, hazardous substance use, and other important military health problems.

## Supplementary material

For supplementary material accompanying this paper visit https://doi.org/10.1017/S0033291720004249. click here to view supplementary material
