# Supplementary material for: Trajectories of functioning in a population-based sample of veterans: contributions of moral injury, PTSD, and depression
Source: Psychol Med. 2020 Nov 25;52(12):2332–41. doi: 10.1017/S0033291720004249 (PMC9527674; doi:10.1017/S0033291720004249)
Supplement: Supplementary file 1 [file S0033291720004249sup001.zip › S0033291720004249sup001.docx]

Supplemental Table 1. Descriptive Statistics and Bivariate Associations among Study Variables (*N* = 7,200)

| Variable | 1. | 2. | 3. | 4. | 5. | 6. | 7. | 8. | 9. | 10. | 11. | 12. | 13. | 14. | 15. | 16. |
| --- | --- | --- | --- | --- | --- | --- | --- | --- | --- | --- | --- | --- | --- | --- | --- | --- |
| 1. Work Functioning | - |  |  |  |  |  |  |  |  |  |  |  |  |  |  |  |
| 2. Health Functioning | .42* | - |  |  |  |  |  |  |  |  |  |  |  |  |  |  |
| 3. Int. Relationship Functioning | .43* | .57* | - |  |  |  |  |  |  |  |  |  |  |  |  |  |
| 4. Posttraumatic Stress | -.17* | -.21* | -.21* | - |  |  |  |  |  |  |  |  |  |  |  |  |
| 5. Depressive Symptoms | -.38* | -.53* | -.49* | .32* | - |  |  |  |  |  |  |  |  |  |  |  |
| 6. Moral Injury – Witness | -.20* | -.24* | -.22* | .19* | .33* | - |  |  |  |  |  |  |  |  |  |  |
| 7. Moral Injury – Perpetration | -.26* | -.29* | -.27* | .19* | .33* | .57* | - |  |  |  |  |  |  |  |  |  |
| 8. Moral Injury - Betrayal | -.24* | -.32* | -.29* | .21* | .40* | .62* | .54* | - |  |  |  |  |  |  |  |  |
| 9. Female (ref. Male) | -.03 | .07* | .07* | .03 | .07* | .10* | .02 | .11* | - |  |  |  |  |  |  |  |
| 10. Age | .03 | .11* | -.09* | .06* | -.08* | -.06* | -.12* | -.05* | -.08* | - |  |  |  |  |  |  |
| 11. Minority Racial Status (ref. White) | -.06* | -.10* | -.09 | .06* | .12* | .12* | .08* | .11* | .11* | -.07* | - |  |  |  |  |  |
| 12. Navy (ref. Army) | .08* | .04* | .06* | -.06* | -.05* | -.02 | .01 | -.02 | .04* | .01 | -.06* | - |  |  |  |  |
| 13. Air Force (ref. Army) | .11* | .15* | .13* | -.07* | -.14* | -.12* | -.10* | -.10* | .05* | .09* | -.12* | - | - |  |  |  |
| 14. Marine (ref. Army) | .06* | .01 | .05* | -.01 | -.04 | -.06* | -.01 | -.06* | -.04* | -.11* | -.05* | - | - | - |  |  |
| 15. Officer (ref. Enlisted) | .08* | .24* | .08* | -.04 | -.17* | -.07* | -.12* | -.10* | .01 | .39* | -.17* | .01 | .03 | -.07* | - |  |
| 16. Number of Deployments | -.03 | -.01 | -.13* | .11* | .02 | -.01 | -.01 | .01 | -.19* | .52* | -.05* | .01 | .01 | -.06* | .14* | - |
| *Mean* | 4.50 | 3.99 | 3.95 | 0.39 | 1.21 | 2.65 | 1.74 | 2.27 | 18% | 34.23 | 34% | - | - | - | 24% | 1.41 |
| *Std. Deviation* | 0.62 | 0.55 | 0.83 | 1.16 | 1.67 | 1.64 | 1.21 | 1.47 | - | 9.52 | - | - | - | - | - | 1.17 |

Note. Sociodemographic and military-related characteristics were coded as follows: female (male = 0, 1 = female), age (continuous years), minority racial status (white = 0, non-white = 1), navy (army = -1, other = 0, navy = 1), air force (army = -1, other = 0, air force = 1), marine (army = -1, other = 0, marine = 1), officer (enlisted = 0, officer = 1), and number of deployments (none = 0, one = 1, two = 2, three or more = 3). **p < .001*
