# Supplementary figures and images for: Trajectories of functioning in a population-based sample of veterans: contributions of moral injury, PTSD, and depression
Source: Psychol Med. 2020 Nov 25;52(12):2332–41. doi: 10.1017/S0033291720004249 (PMC9527674; doi:10.1017/S0033291720004249)

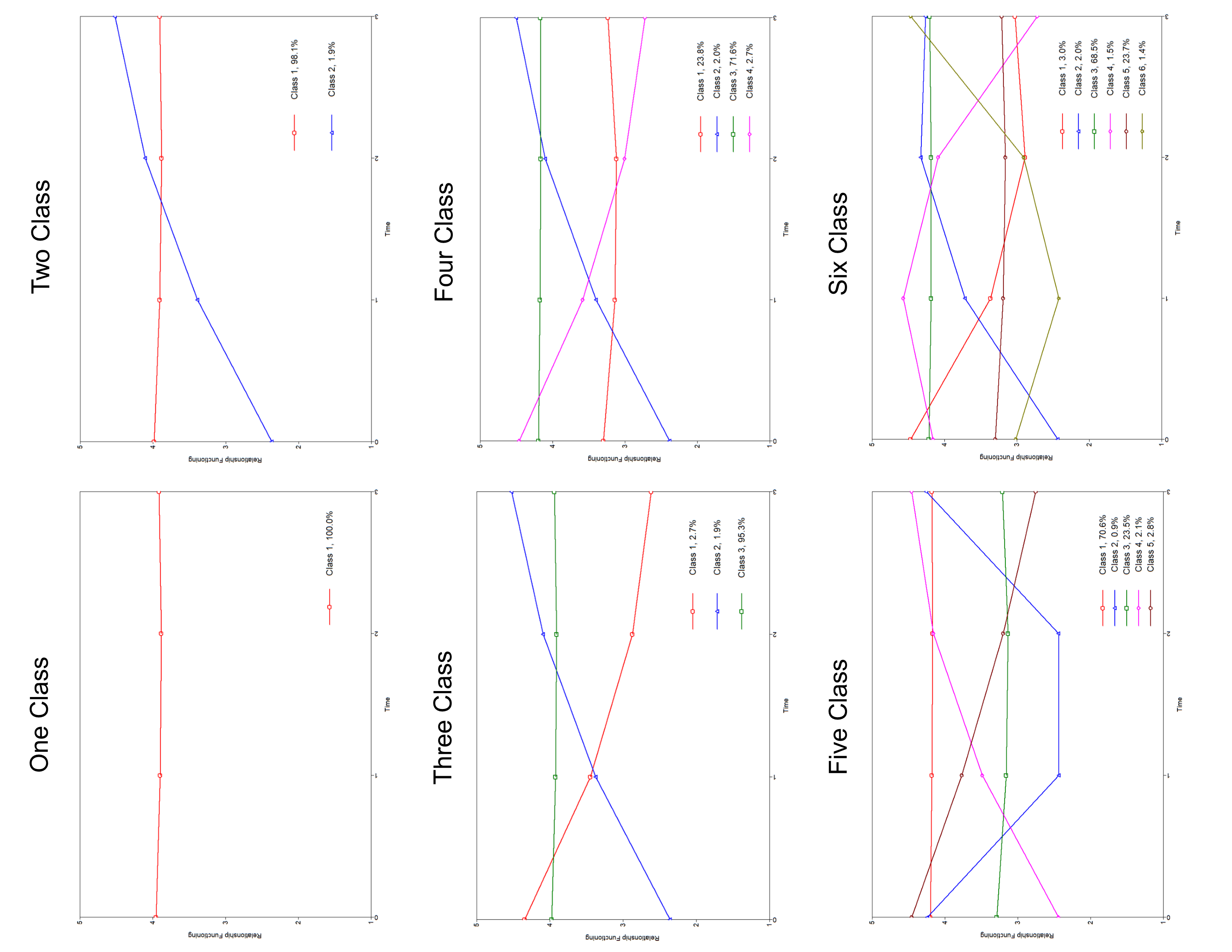

Supplement: Supplementary file 1 [file S0033291720004249sup001.zip › S0033291720004249sup002.tif]

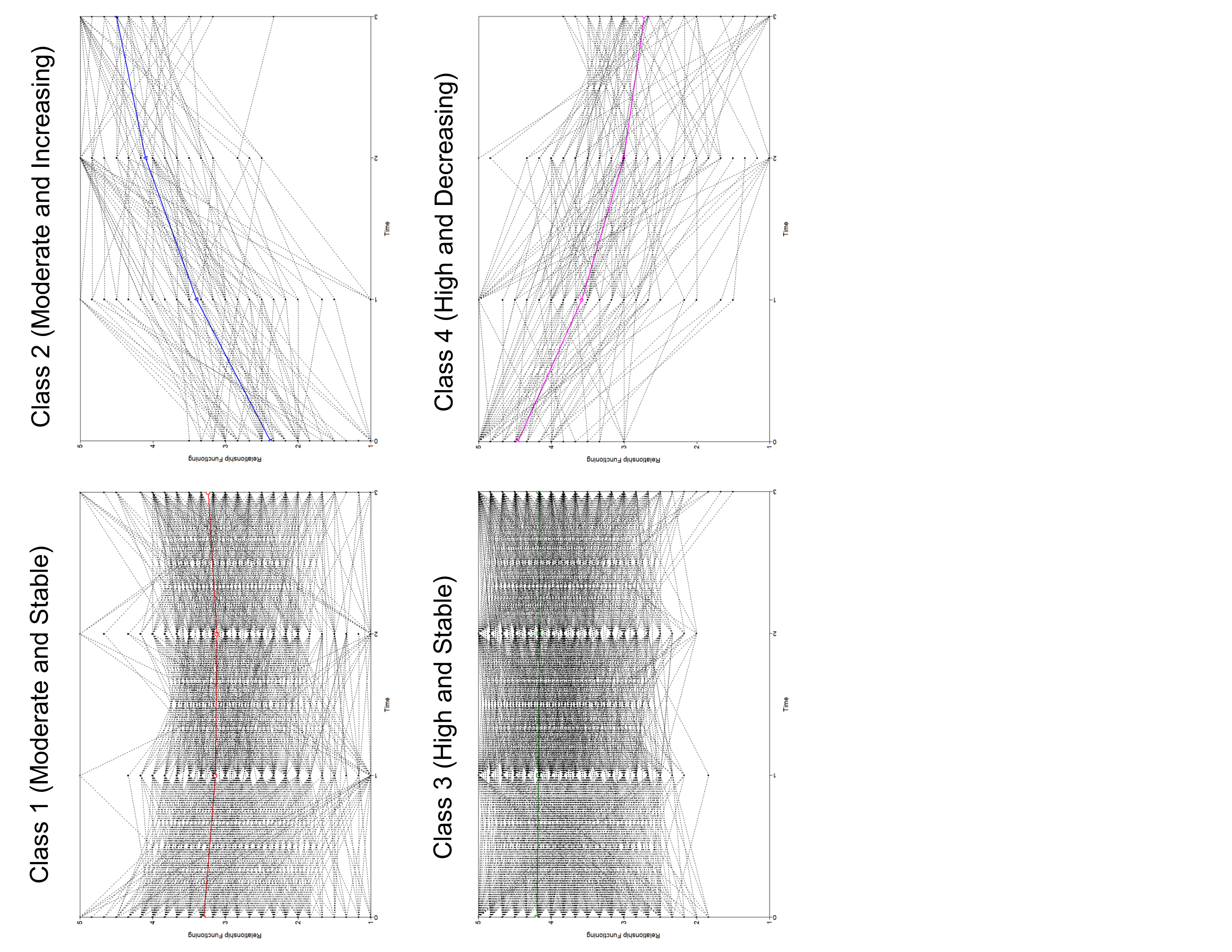

Supplement: Supplementary file 1 [file S0033291720004249sup001.zip › S0033291720004249sup003.tif]

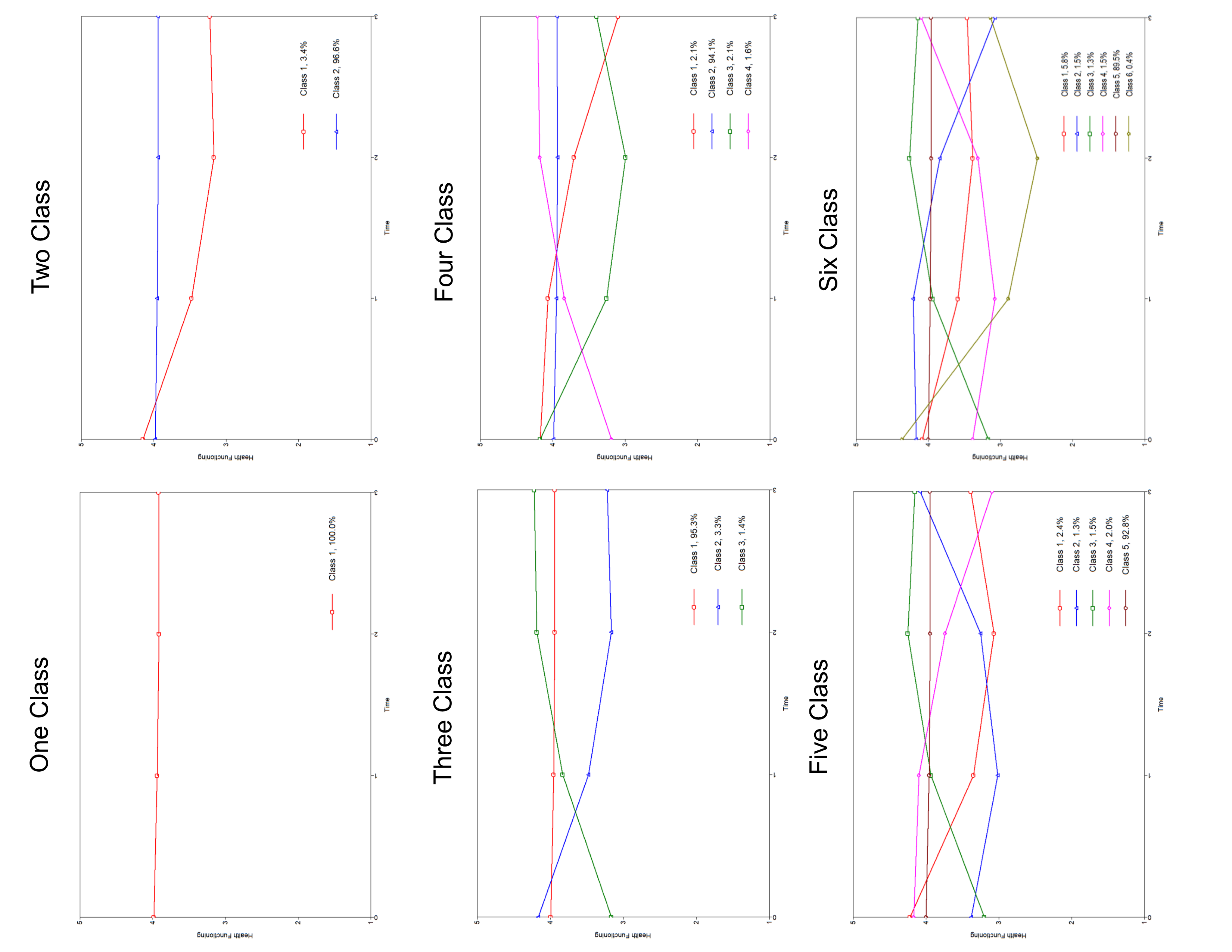

Supplement: Supplementary file 1 [file S0033291720004249sup001.zip › S0033291720004249sup004.tif]

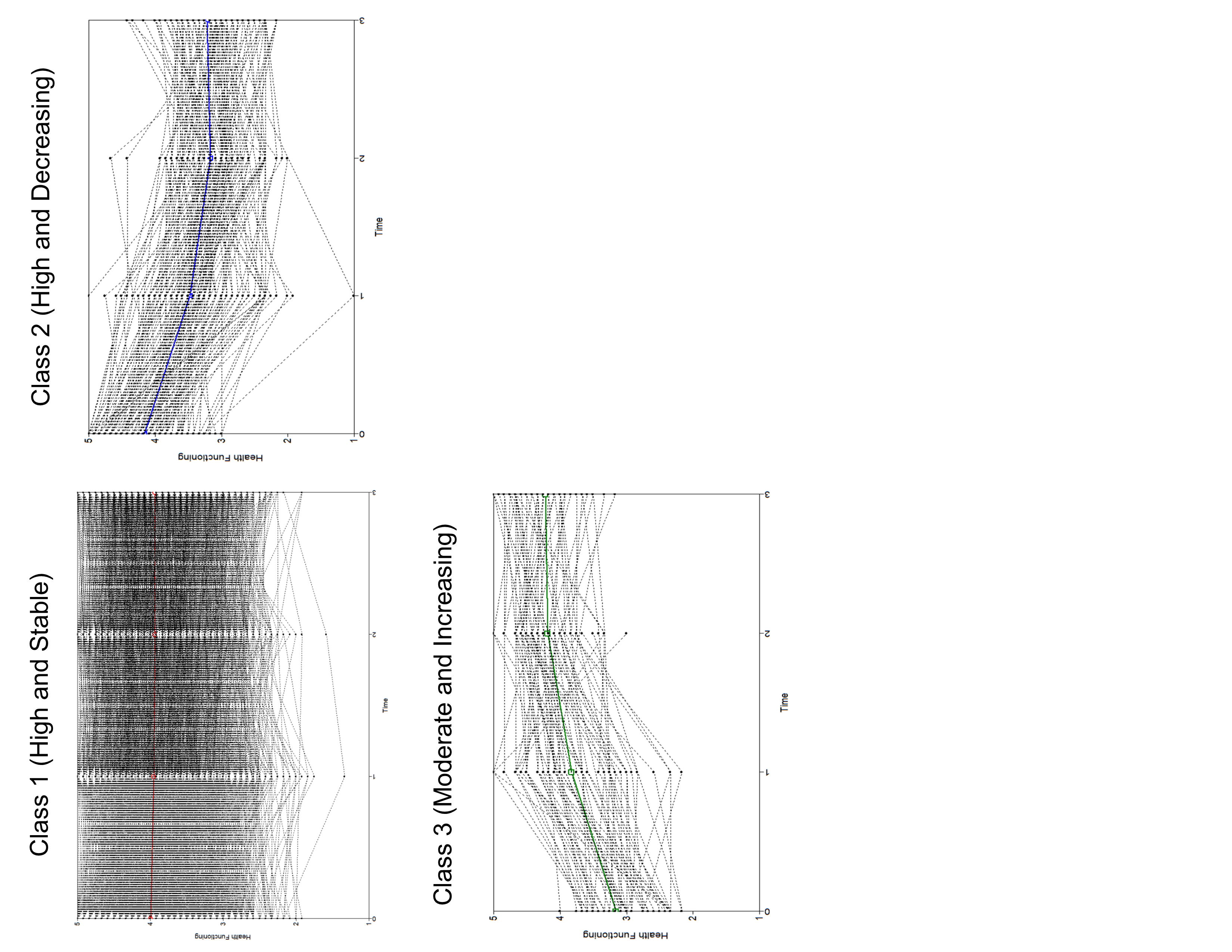

Supplement: Supplementary file 1 [file S0033291720004249sup001.zip › S0033291720004249sup005.tif]

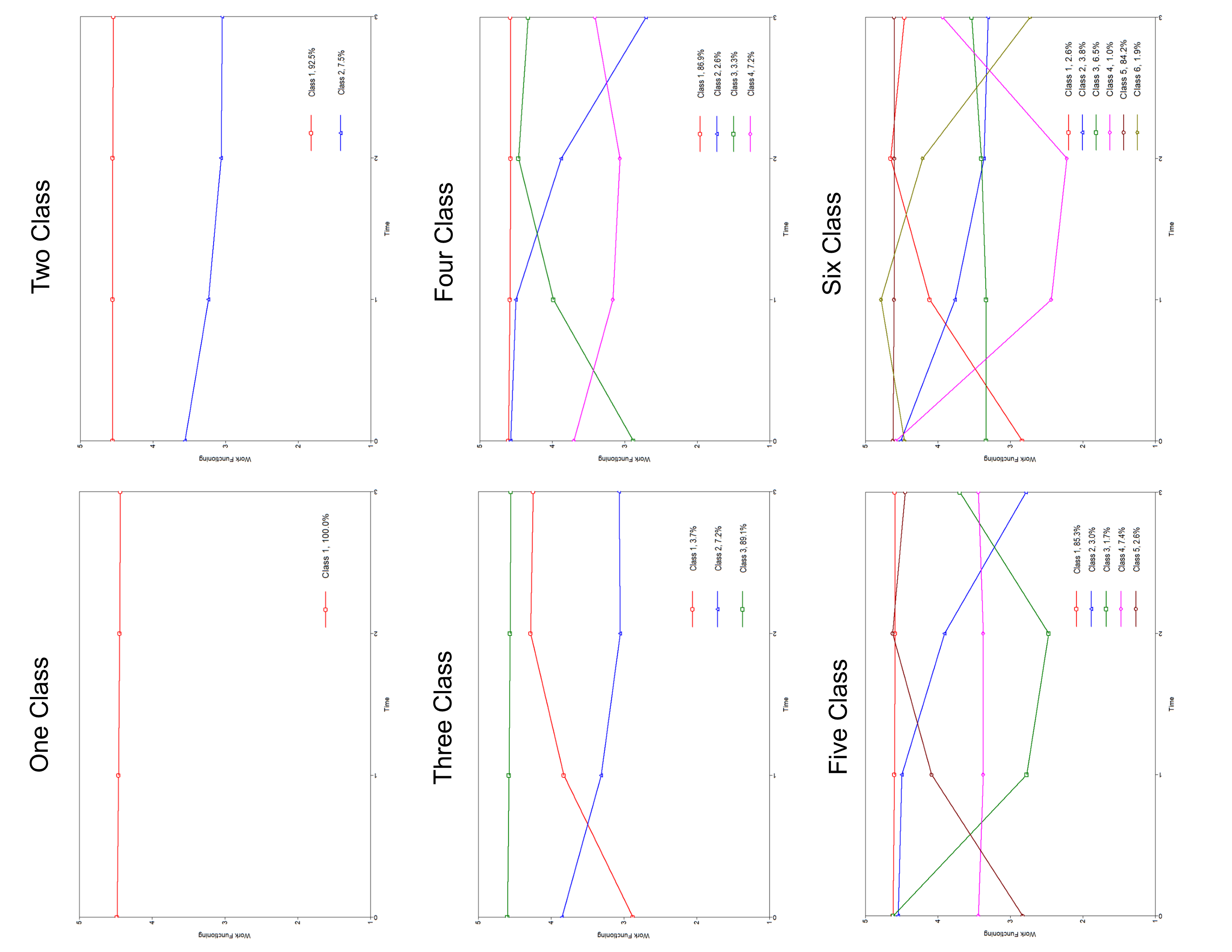

Supplement: Supplementary file 1 [file S0033291720004249sup001.zip › S0033291720004249sup006.tif]

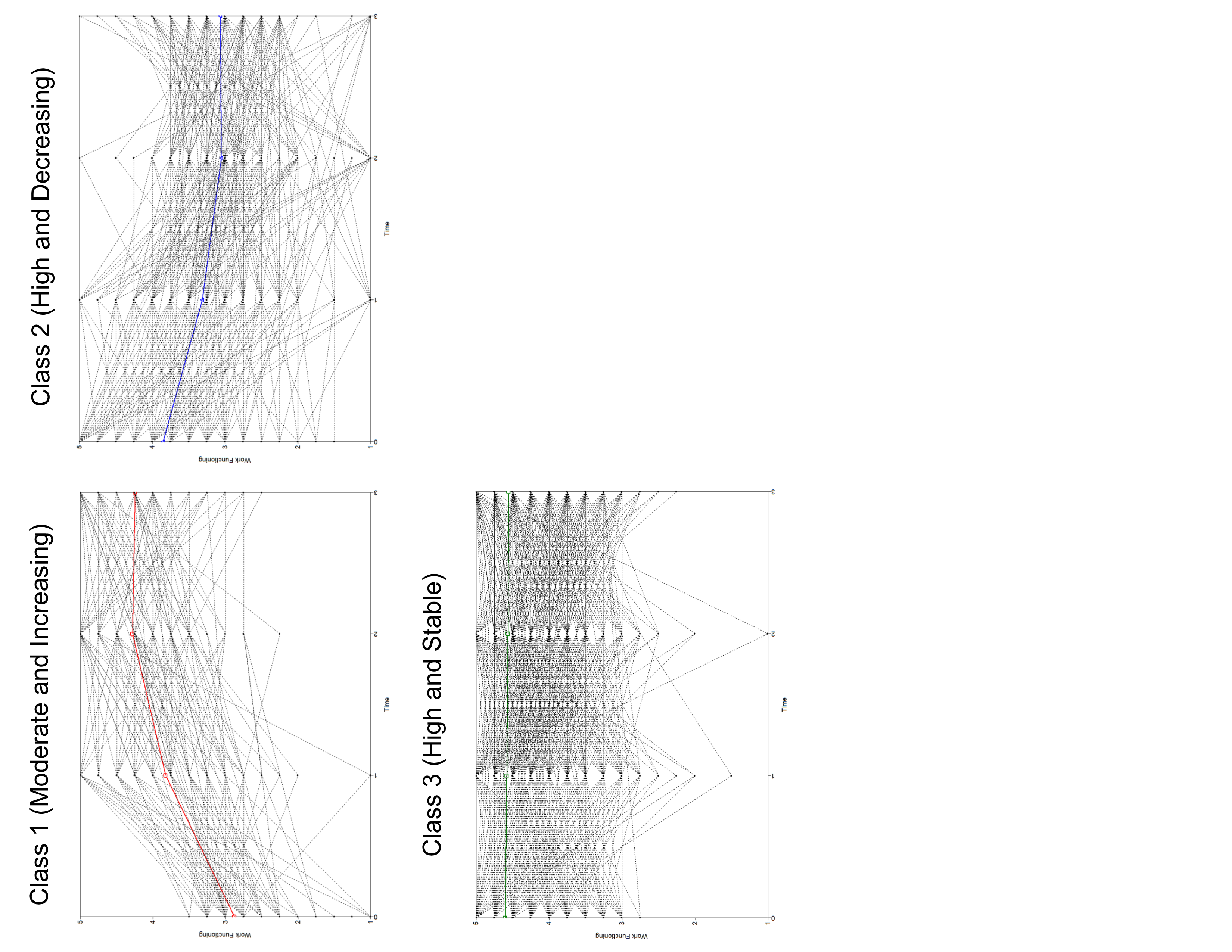

Supplement: Supplementary file 1 [file S0033291720004249sup001.zip › S0033291720004249sup007.tif]
